# XE991 Reverses Enhanced Short-Term Plasticity in Rat Epileptic CA1

**DOI:** 10.3390/brainsci16090929

**Published:** 2026-08-31

**Authors:** Emma Schnell, Steffen Müller, Rüdiger Köhling, Timo Kirschstein

**Affiliations:** 1 Oscar Langendorff Institute of Physiology, University Medicine Rostock, Gertrudenstrasse 9, 18057 Rostock, Germany; emma.schnell@uni-rostock.de (E.S.); steffen.mueller@hmu-erfurt.de (S.M.); ruediger.koehling@uni-rostock.de (R.K.); 2 Medical Faculty, HMU Health and Medical University Erfurt, Dalbergsweg 9, 99084 Erfurt, Germany

**Keywords:** Schaffer collateral, excitatory postsynaptic potential, KCNQ

## Abstract

**Background/Objectives**: Recent evidence has suggested a transcriptional reduction in Kv7 potassium channels in the CA1 area of acquired experimental epilepsy, but presynaptic Kv7 channels might be spared. Here, we tested the influence of the Kv7 channel blocker XE991 on two forms of short-term plasticity, which are supposed to be presynaptic in nature: post-tetanic potentiation and paired-pulse ratio. **Methods**: Systemic pilocarpine was administered to induce status epilepticus and chronic epilepsy in the rat. In the chronically epileptic CA1 area, extracellular electrophysiological experiments were performed to analyze paired-pulse ratio before and after tetanic stimulation (100 Hz, 1 s). **Results**: Both the post-tetanic potentiation and the paired-pulse ratio were significantly higher in the epileptic CA1 as compared to control tissue (Cohen’s d > 3; *n* = 10–11 slices from 3–4 animals per group). While pharmacological inhibition of Kv7 channels with XE991 had no effect on these measures in control CA1, it significantly reversed both forms of enhanced short-term plasticity in the epileptic CA1 to control levels (Cohen’s d > 3; *n* = 10–11 slices from 3 animals per group). **Conclusions**: The epileptic CA1 showed enhanced short-term plasticity, which was not due to the loss of Kv7 channels in the epileptic CA1 subfield, since it was reversed by XE991. Therefore, our findings provide indirect evidence that the presynaptic Kv7 channel function may be at least partially intact in the epileptic CA1. It may be speculated whether potassium channel activators could pose a risk to enhance rather than depress short-term plasticity at Schaffer collateral–CA1 synapses in epilepsy.

## 1. Introduction

Voltage-dependent potassium channels of the Kv7 group (also known as KCNQ channels) constitute non-inactivating K^+^ currents and are powerful regulators of neuronal excitability [1,2,3,4]. Given this physiological role in hyperpolarizing the resting membrane potential and shaping the afterpotential following action potentials [1,2,3,4], Kv7 channel inhibition was expected to enhance long-term potentiation (LTP). Indeed, XE991-induced LTP facilitation in CA1 pyramidal cells was found using both theta-burst [4] and tetanic stimulation protocols [5,6]. In the dentate gyrus, there is only one study that tested co-application of XE991 and tolbutamide and also reported an LTP accentuation as a consequence of Kv7 and Kir6 channel inhibition [7]. Taken together, the available literature points to a dampening role of Kv7 channels in LTP, which is well consistent with the predominantly postsynaptic nature of LTP expression in CA1 [8], but also with the postsynaptic location of Kv7 channels on CA1 dendrites [4,9]. However, one has to bear in mind that the hippocampal subunits of Kv7 channels, Kv7.2 and Kv7.3, were not only localized to post- but also to presynaptic sites [10,11,12,13], and activation of presynaptic Kv7 channels was associated with reduced glutamate and GABA release [14]. Hence, both pre- and postsynaptic Kv7 channels are powerful regulators of excitability of excitatory as well as inhibitory neurons.

Mutations of the hippocampal Kv7 subunits, Kv7.2 and Kv7.3, are well-known genetic causes of infantile epilepsies [15,16]. But more importantly, both subunits were also found to be downregulated in acquired temporal lobe epilepsy in rats [17,18,19], as well as in humans [20]. In line with this, we demonstrated that XE991 failed to aggravate disinhibition-induced hyperexcitability in the chronically epileptic CA1 [19]. In the same study, we also found that XE991 still reduced the paired-pulse ratio, suggesting that presynaptic Kv7 channels might have been spared [19]. Moreover, the frequency of spontaneous excitatory currents triggered by gabazine and hyperkalemia was enhanced by XE991, and this effect was significantly more pronounced in epileptic CA1 neurons [21]. These findings raise the question whether presynaptic Kv7 channels may shape transmitter release from Schaffer collaterals and, in particular, how this shaping might be altered in epileptic tissue.

Here, we asked the question of how XE991 affects two types of short-term plasticity, paired-pulse ratio (PPR) and post-tetanic potentiation (PTP), in the pilocarpine model of chronic epilepsy. In line with our hypothesis that short-term plasticity should be enhanced due to the lack of Kv7 channel function, we found increased PTP and PPR in the epileptic CA1. However, XE991 reversed both types of short-term plasticity to control levels, and it had no significant effect on control tissue. These findings suggest that presynaptic Kv7 channels had shaped transmitter release in epileptic tissue.

## 2. Materials and Methods

### 2.1. Pilocarpine-Induced Status Epilepticus

Status epilepticus (SE) was induced as described [19]. Male Wistar rats (100 ± 10 g, 30 ± 1 days old, mean ± S.D.; Charles River, Sulzfeld, Germany) were pre-treated with N-methylscopolamine (1 mg/kg i.p.; Sigma, Taufkirchen, Germany) to dampen peripheral cholinergic effects [22], and after 30 min, they were injected either with pilocarpine hydrochloride (340 mg/kg i.p.; Sigma) or saline. Following pilocarpine administration, rats developed spontaneous convulsions with gradual progression into SE. We determined the onset of SE as a stage 4 or 5 seizure [23], followed by continuous motor seizures without showing any reaction to sensory stimuli, such as gently touching the whiskers. SE was allowed to last for 40 min before all convulsions were suppressed by diazepam (4 mg/kg i.p., repeated as needed). To promote recovery from SE, animals were repeatedly fed with 5% glucose solution for 24 h. All experiments were performed in rats at 9 ± 5 weeks after pilocarpine/saline treatment (mean ± S.D). Thus, all pilocarpine-treated animals presented at least one seizure before being used in this study. Since epileptic animals are commonly more sensitive to the experimenter’s approach, animals were not used in a blinded fashion.

All animal procedures were carried out according to national and international guidelines on the ethical use of animals (European Council Directive 2010/63/EU) and approved by the “Landesamt für Landwirtschaft, Lebensmittelsicherheit und Fischerei Mecklenburg-Vorpommern” (protocol code 7221.3-1.1-054/18). We confirm that all efforts were made to minimize animal suffering and to reduce the number of animals used.

### 2.2. Extracellular Electrophysiology

Rats were deeply anesthetized with diethyl ether, confirmed by loss of the righting reflex and loss of the pedal withdrawal reflex. Then, brains were rapidly removed and submerged into oxygenated ice-cold sucrose-based dissection solution containing (in mM) 87 NaCl, 75 sucrose, 25 NaHCO_3_, 2.5 KCl, 1.25 NaH_2_PO_4_, 0.5 CaCl_2_, 7 MgCl_2_, and 10 D-glucose (95% O_2_, 5% CO_2_; pH 7.4; 326–328 mosmol/kg). Unless otherwise indicated, chemicals were purchased from Sigma-Aldrich (Taufkirchen, Germany). Horizontal hippocampal slices of 400 µm thickness were cut using a vibratome (Integraslice 7550MM, Campden Instruments, Loughborough, UK) and stored in artificial cerebrospinal fluid (ACSF) containing (in mM) 125 NaCl, 26 NaHCO_3_, 3 KCl, 1.25 NaH_2_PO_4_, 2.5 CaCl_2_, 1.3 MgCl_2_, and 13 D-glucose (306–314 mosmol/kg, bubbled with 95% O_2_ and 5% CO_2_ to maintain the pH at 7.4).

Extracellular recordings were undertaken in a Haas-type interface chamber (flow rate of 2 mL/min, temperature 32 ± 1 °C, npi electronic GmbH, Tamm, Germany). We recorded field excitatory postsynaptic potentials (fEPSP) in the CA1 stratum radiatum using borosilicate glass capillaries (GB150-8P, Science Products, Hofheim, Germany). Signal processing included amplification and filtering at 1 kHz (EXT-10-2F, npi electronic GmbH, Tamm, Germany) and digitization (Micro1401 analog-to-digital converter, Signal 2.16 software, Cambridge Electronic Design, Cambridge, UK). Constant-current mode stimulation (A365, World Precision Instruments, Sarasota, FL, USA) of Schaffer collaterals was achieved using bipolar electrodes (PT-2T, Science Products, Hofheim, Germany) placed into the stratum radiatum at the border between CA2 and CA1.

Baseline stimulation intensity was adjusted to elicit an fEPSP with 40–50% of its maximum amplitude. Following 5 min of baseline stimulation (double pulses with an inter-pulse interval of 40 ms every 30 s, controlled by Master-8; A.M.P.I., Jerusalem, Israel), we applied a tetanic stimulation protocol (100 Hz for 1 s) at double baseline stimulation intensity, and then baseline stimulation (double pulses) was delivered again for 10 min follow-up. To study the influence of Kv7 channels, we used 10 µM XE991 (Tocris, Bristol, UK) as before [19]. Since epileptic discharges developed over 30 min in that study [19], we decided to pre-incubate the slices for 60 min with 10 µM XE991 prior to the transfer into the recording chamber. From all animals, we assigned half of the slices to XE991 pre-incubation, while the remainder was maintained in standard ACSF. The order of slices to be recorded from was randomized.

Since XE991 often caused repetitive afterpotentials [19], we always obtained the initial negative slope of the fEPSP rather than its peak amplitude. All fEPSP slopes were then normalized to the individual baseline value and expressed as a percentage of baseline (termed “% of pre”). For further analyses, three timepoints were selected: a pre-tetanic period of 2.5 min (mean of 5 values) directly before tetanic stimulation (referred to as “timepoint pre”), the timepoint of PTP (mean of 2 values = 1 min after tetanic stimulation, referred to as “timepoint ptp”), and a follow-up period of 2.5 min at the end of the experiment (mean of 5 values, referred to as “timepoint post”). The paired-pulse ratio (PPR) was likewise calculated as the ratio of the second fEPSP slope divided by the first fEPSP slope. Absolute PPR values are shown as decimal numbers without unit; relative PPR values (normalized to pre-tetanic baseline values) are shown as percentages with the unit “% of pre”.

### 2.3. Statistics

Numerical data in the text were expressed as mean values ± SEM. All *n*-numbers refer to slice numbers, but animal numbers are also given. We performed all statistical analyses in a slice-based and an animal-based fashion for the following reasons: On the one hand, the XE991 effect was tested on slices after brain preparation, making them independent statistical units. However, it cannot be excluded that all slices from a given animal may be inherently correlated. On the other hand, with respect to animal welfare, in particular in the case of pilocarpine-treated animals, it is unethical to record only one or two slices per animal.

Hence, we had four experimental groups: (1) slices from saline-treated animals without XE991 (“Ctrl”), (2) slices from saline-treated animals incubated with XE991 (“Ctrl-XE”), (3) slices from pilocarpine-treated animals without XE991 (“Epil”), and (4) slices from pilocarpine-treated animals incubated with XE991 (“Epil-XE”). To analyze the effects of XE991 and the tetanic stimulation, respectively, the following statistical tests were used (SigmaPlot 13.0 software): (i) The 2-way analysis of variance (2-way-ANOVA) followed by the Holm–Sidak post hoc test assessed the influence of two factors and their interaction (factor animal, i.e., “Ctrl” versus “Epil”, and factor drug, i.e., “XE” versus “no XE”) and was carried out in both the slice-based as well as the animal-based analysis. (ii) The Friedman repeated measures ANOVA on ranks followed by Tukey’s post hoc test assessed the influence of tetanic stimulation on the PPR and was included in the slice-based analysis. All given *p* values refer to the post hoc test of the slice-based analysis. For the sake of clarity, Table 1 gives all *p* values for both the slice-based and the animal-based analyses, respectively (2-way ANOVA).

The animal-based analysis also included Cohen’s d as a measure of effect size given for the most relevant findings. Cohen’s d was calculated as follows:

$$Cohen’s\;d\;=\;\frac{{Mean}_{1}-{Mean}_{2}}{\sqrt{\frac{\left(n_{1}-1\right)\times {Var}_{1}+(n_{2}-1)\times {Var}_{2}}{\left(n_{1}+n_{2}-2\right)}}}$$

where *Mean*, *Var*, and *n* refer to the arithmetic mean, the variance, and the animal number, respectively, of both data sets to be compared (i.e., “Ctrl” versus “Epil” or “Epil” versus “Epil-XE”).

## 3. Results

**Figure 1:**
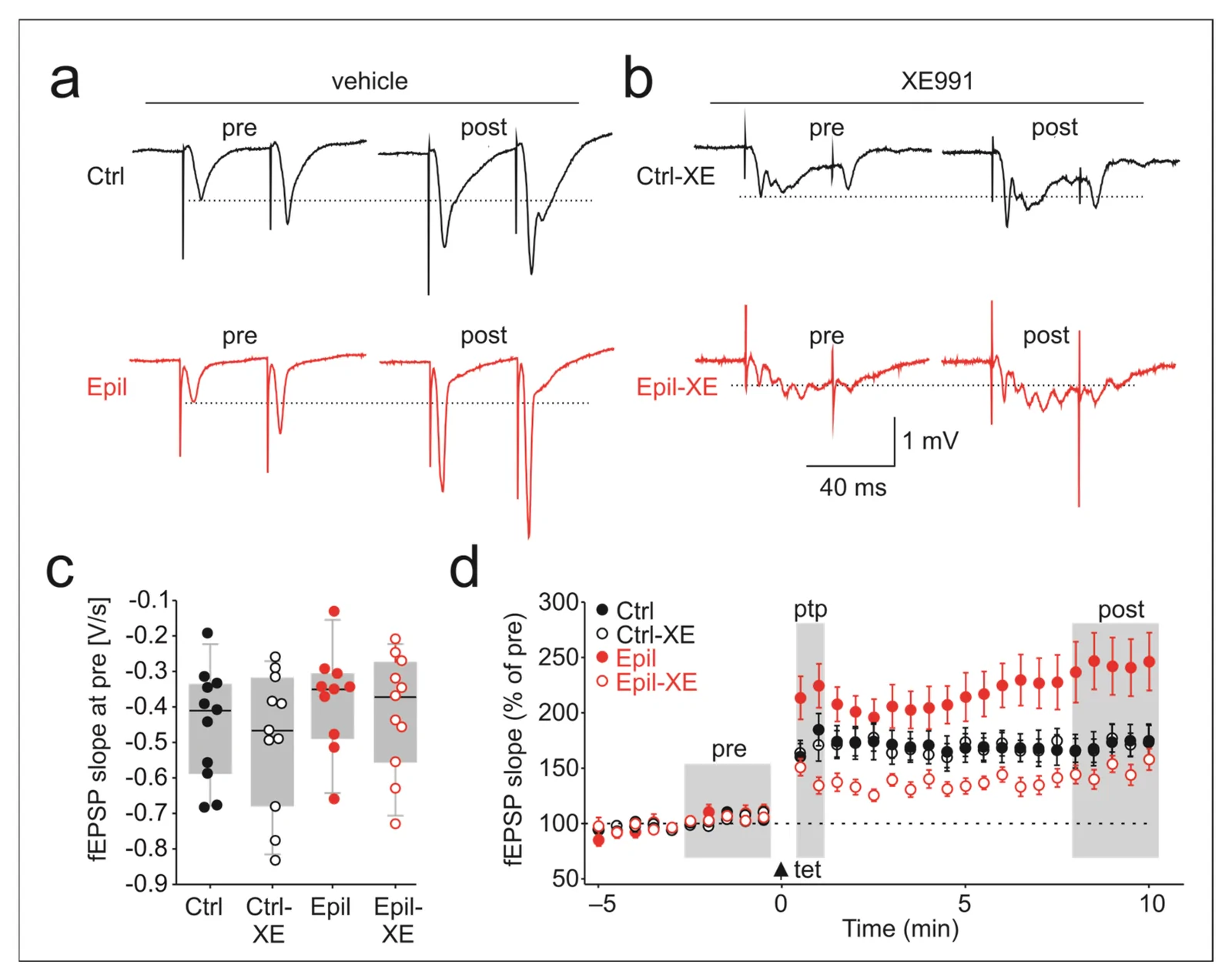
(**a**,**b**) Representative fEPSP recordings from control (“Ctrl” and “Ctrl-XE”, black traces) and chronically epileptic CA1 (“Epil” and “Epil-XE”, red traces) directly before tetanic stimulation (pre) and after 10 min of follow-up (post). The dotted line indicates the amplitude of the fEPSP before tetanic stimulation in all four sample recordings. (**b**) Note that XE991 (“Ctrl-XE” and “Epil-XE”) caused repetitive postsynaptic potentials in both animal groups [19]. (**c**) Absolute fEPSP slope values at baseline for all four experimental groups. The box-and-whisker plots indicate the median (black line), the first and third quartile (gray box), and the 10th and 90th percentile (gray whiskers). All symbols refer to slices. There were no significant differences among all four groups (Ctrl: *n* = 11 slices from four animals, Ctrl-XE: *n* = 11 slices from four animals, Epil: *n* = 10 slices from three animals, Epil-XE: *n* = 11 slices from three animals). (**d**) Relative fEPSP slope (expressed as mean percentage of baseline values ± SEM) before (negative timepoints) and after tetanic stimulation (positive timepoints). Tetanic stimulation is indicated by an arrow and “tet”. The gray boxes indicate the timepoints pre, ptp, and post. The two-way ANOVA (factors animal and drug) found a significant difference between “Epil” and “Ctrl” both at the timepoint ptp (*p* < 0.05) and at the timepoint post (*p* < 0.01). The dotted line indicates 100% of pre.

The aim of this study was to explore the effect of XE991 on short-term plasticity at control and chronically epileptic Schaffer collateral–CA1 synapses. Figure 1a shows representative field excitatory postsynaptic potentials (fEPSPs) from control and epileptic CA1 directly before (timepoint pre) and 10 min after (timepoint post) tetanic stimulation. Directly after tetanic stimulation (timepoint ptp) and after 10 min of follow-up, fEPSP slopes were significantly higher in epileptic as compared to control slices (ptp: 219 ± 19% of pre, *n* = 10 slices from three animals versus 173 ± 12% of pre, *n* = 11 slices from four animals; post: 242 ± 26% of pre, *n* = 10 versus 171 ± 15% of pre, *n* = 11, *p* < 0.05 for both, two-way ANOVA with the Holm–Sidak post hoc test, Figure 1d, Table 1). The effect sizes based on animal numbers for both measures were 3.3 and 3.2, respectively, indicating a strong effect of SE on CA1 potentiation. When XE991 was present in the bath (Figure 1b), fEPSPs tended to have smaller amplitudes and to show repetitive epileptiform afterpotentials as previously described [19]. In fact, we detected a significant group difference of fEPSP amplitudes between control (“no XE”: −1.02 ± 0.09 mV, “XE”: −1.00 ± 0.07 mV) and epileptic slices (“no XE”: −0.70 ± 0.09 mV, “XE”: −0.65 ± 0.07 mV; *p* < 0.001 for “Ctrl” versus “Epil”, two-way ANOVA with the Holm–Sidak post hoc test, Table 1), similar to our previous study [19]. However, the fEPSP slope was comparable in all groups (Figure 1c), indicating that the amplitude had been truncated by early epileptiform afterpotentials.

In the presence of XE991, Schaffer collateral–CA1 synapses from control animals showed normal potentiation levels (ptp: fEPSP slopes 167 ± 12% of pre, post: 171 ± 11% of pre, *n* = 11), while in epileptic tissue potentiation was dramatically reduced (ptp: fESPSP slopes 142 ± 6% of pre, post: 148 ± 8% of pre, *n* = 11, *p* < 0.001 for both timepoints ptp and post versus epileptic CA1 without XE991, two-way ANOVA with the Holm–Sidak post hoc test, Figure 1d, Table 1). Again, the effect sizes based on animal numbers for both measures were 3.6 and 3.5, respectively, indicating a strong effect of XE991 on epileptic CA1. Hence, these data supported our hypothesis of enhanced potentiation in epileptic CA1, but this finding can hardly be attributed to impaired Kv7 channel expression in this tissue. In addition, our data also suggest that Kv7 channel function plays a minor role in shaping potentiation under control conditions.

**Figure 2:**
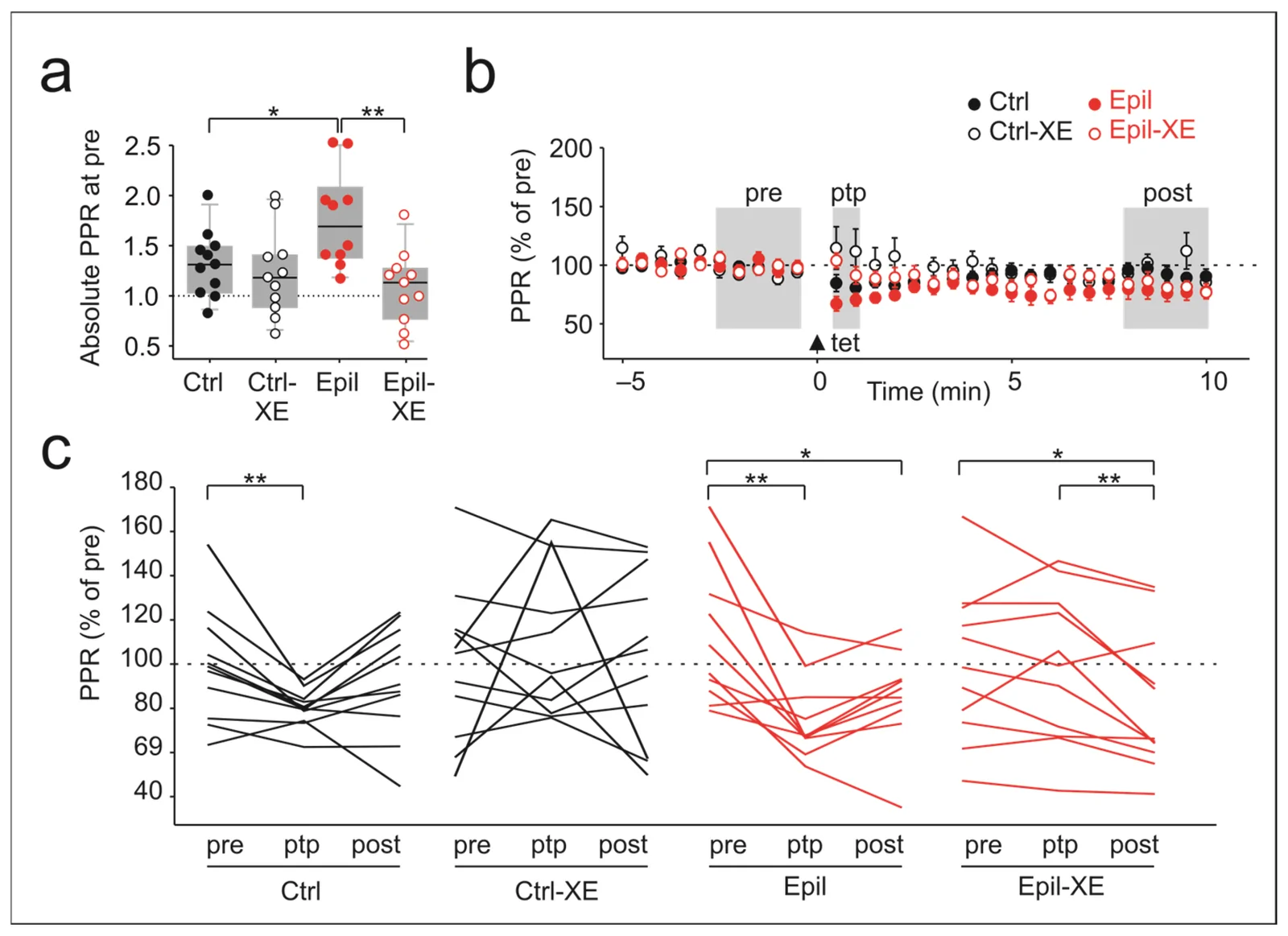
(**a**) Absolute paired-pulse ratios (PPR) of all slices at timepoint pre. The box-and-whisker plots indicate the median (black line), the first and third quartile (gray box), and the 10th and 90th percentile (gray whiskers). There were significant differences between “Ctrl” and “Epil” and between “Epil” and “Epil-XE” (* *p* < 0.05, ** *p* < 0.01, 2-way ANOVA, factors animal and drug, with the Holm–Sidak post hoc test). (**b**) Time course of the relative PPR (in % of pre) expressed as the mean percentage (±SEM). Tetanic stimulation is indicated by an arrow and “tet”. The gray boxes indicate the timepoints pre, ptp, and post. The dashed line indicates 100% of pre. (**c**) Relative PPR (in % of pre) of all individual slices at timepoints pre, ptp, and post. The PPR dropped significantly directly after tetanic stimulation in slices without XE991 only (“Ctrl” and “Epil”), but not in XE991-treated slices. PPR changes at timepoint post compared to that at timepoint pre were only found in epileptic slices (* *p* < 0.05, ** *p* < 0.01, Friedman repeated measures ANOVA on ranks with Tukey’s post hoc test).

Since our previous study demonstrated that XE991 reduced the paired-pulse ratio [19], we analyzed the paired-pulse ratio (PPR) in the present study, too. Whereas there was only a slight reduction in the PPR in control slices when XE991 was present (“no XE”: 1.31 ± 0.10, *n* = 11; “XE”: 1.14 ± 0.11, *n* = 11), the PPR in epileptic slices without XE991 was significantly higher than in control (1.73 ± 0.15, *n* = 10, *p* < 0.05 versus “Ctrl”, 2-way ANOVA with the Holm–Sidak post hoc test, Table 1), but more importantly, XE991 significantly depressed this PPR in epileptic slices (1.07 ± 0.11, *n* = 11, *p* < 0.001 versus “Epil”, 2-way ANOVA with the Holm–Sidak post hoc test, Figure 2a, Table 1), resulting in values comparable to control slices. Here, the effect sizes based on animal numbers for both findings were 3.2 and 5.2, respectively, indicating a strong effect of SE on PPR and likewise XE991 on PPR in epileptic tissue. Moreover, tetanic stimulation did not alter PPR in control slices (“no XE”: 93 ± 7% of pre, “XE”: 104 ± 11% of pre) but led to a substantial PPR drop in both groups of epileptic slices (“no XE”: 76 ± 6% of pre, “XE”: 83 ± 9% of pre, Figure 2b). Data from the individual experiments depicted in Figure 2c confirmed significant differences between pre and post values in epileptic CA1 and showed further significant differences between timepoints pre and ptp in non-XE991 slices and between ptp and post in XE991-treated epileptic slices (Friedman repeated measures ANOVA on ranks with Tukey’s post hoc test).

In summary, the PPR data showed that XE991 normalized the enhanced PPR in non-potentiated epileptic tissue, corroborating the PTP data as shown above. In addition, they also showed that tetanic stimulation led to persistent PPR changes in epileptic tissue, only suggesting that presynaptic mechanisms had been at least partially involved. Together with the PTP data, our present findings support the view of presynaptic Kv7 channel function at Schaffer collateral–CA1 synapses in chronically epileptic tissue. Given that Kv7 channel inhibition reduced the enhanced short-term plasticity in epileptic CA1, our findings also may generate the hypothesis that potassium channel openers could enhance short-term plasticity and probably epileptic activity.

## 4. Discussion

The aim of the present study was to explore short-term plasticity in the chronically epileptic CA1 region. Here, we analyzed two commonly used forms of short-term plasticity: the post-tetanic potentiation (PTP) and the paired-pulse ratio (PPR). Our major findings were (1) that epileptic tissue exhibited an enhancement of both forms of short-term plasticity and (2) that the Kv7 channel blocker XE991 reversed the epilepsy-related enhancement of short-term plasticity. Since previous reports had observed enhanced long-term potentiation at epileptic Schaffer collateral–CA1 synapses [24,25,26], we anticipated an enhanced PTP at epileptic synapses, which proved true. Likewise, our previous study had shown slightly higher PPR in epileptic tissue, but without reaching statistical significance [19]. Here, we repeated this experiment and showed that the PPR using fEPSP slopes rather than amplitudes was also enhanced in epilepsy as compared to control, corroborating previous findings in the lithium-pilocarpine model of epilepsy [27]. Provided that Kv7.2 and Kv7.3 channels were reduced in epileptic tissue [17,18,19], we hypothesized that XE991 had less effect on these measures in epileptic tissue. But contrary to this, we obtained an XE991-mediated reversal of the enhanced PTP and PPR in epilepsy back to control levels, which were novel findings. One might assume that the XE991-induced epileptiform afterpotentials could have affected short-term plasticity. In fact, we believe that this possibility is rather unlikely, since XE991 did not alter PTP or PPR in control slices. Furthermore, this particular finding also suggests that Kv7 channels were less important for short-term plasticity in native tissue but provides evidence that the epilepsy-related Kv7 channel reduction was not evenly allocated to all CA1 cell membranes, but it obviously spared those required for PTP or PPR.

More than 30 years ago, both forms of short-term plasticity analyzed here, PTP and PPR, were found to result from a transient increase in residual presynaptic calcium [28,29]. In fact, increased short-term plasticity is a common finding in mice deficient for presynaptic proteins, such as synapsin-I [30], Rab3a [31], Rim1α [32], synaptotagmin IV [33], or Munc13-2 [34], while postsynaptic long-term potentiation remained unaltered, at least in synapsin-I and Munc13-2 deficiency [30,34]. Following this line of evidence, calcium-dependent priming of the synaptic vesicle protein Munc13-1 was demonstrated to be crucial for paired-pulse and frequency facilitation at mossy fibers [35]. Further evidence for the presynaptic nature of short-term plasticity comes from a study on lipopolysaccharide-induced increase of presynaptic L-type calcium channels, which was accompanied by decreased paired-pulse facilitation and PTP [36]. It should be noted that additional presynaptic mechanisms, such as adenylyl cyclase and hyperpolarization-activated cation current activation, have also been proposed—at least for PTP [37].

Despite quite a large supporting body of studies, however, one should bear in mind that the concept of presynaptic calcium is not unequivocal since postsynaptic manipulation of calcium/calmodulin-dependent kinase altered paired-pulse plasticity [38]. Furthermore, theta-burst stimulation of the dentate gyrus elicited radially propagating calcium waves and increased nuclear NK-κB in distant cells, implying that network level-dependent changes occurred [39]. Nonetheless, even though short-term plasticity may not exclusively rely on presynaptic calcium, conditions impairing the presynaptic release machinery will reduce transmitter release probability and thereby lead to increased short-term plasticity. In turn, a reduction in PTP and PPR should imply an enhanced release probability. Importantly, potassium channel blockers, such as tetraethylammonium and 4-aminopyridine, attenuated PTP [37], inferring that the higher potassium channel conductance at rest dampened transmitter release and hence facilitated short-term plasticity. In our case, XE991 reduced PTP in epileptic tissue, but not in controls, which suggests that either Kv7 represents only a minor fraction of presynaptic potassium channels in native tissue. Alternatively, it is tempting to assume that presynaptic Kv7 function could even be more important in epileptic than in control CA1. Certainly, the latter condition would need further experimental confirmation, but it aligns with our finding that the frequency of spontaneous excitatory currents triggered by gabazine and hyperkalemia was enhanced by XE991, and this effect was significantly more pronounced in epileptic CA1 neurons [21]. Since immunohistochemical or transcriptional data may reflect postsynaptic rather than presynaptic structures, our findings are consistent with previous findings mentioned above [17,18,19]. At least on the functional level, the present study adds to the literature that the prevalent presynaptic Kv7 function fell short of the postsynaptic reduction in epileptic CA1. Altogether, we suggest that those Kv7 channels which have escaped the overall Kv7 channel reduction in chronically epileptic tissue fulfill a presynaptic function at the Schaffer collateral–CA1 synapse.

One should take into consideration that presynaptic Kv7 channels may not necessarily be limited to Kv7 channels on axon terminals of Schaffer collaterals. GABAergic neurons also express Kv7 [14,40,41,42] and may shape transmitter release, too, at Schaffer collateral–CA1 synapses: gabazine and bicuculline lead to altered PPR [43] and, even more importantly, sucrose-based preparation solution, which was also used here, leads to more preserved GABAergic inhibition and, as a consequence of this, reduced PTP [44]. Thus, in our hands, GABAergic interneurons certainly influenced synaptic transmission, and Kv7 channels on these neurons were also XE991 targets. Although the initial negative fEPSP slope is less contaminated by GABAergic input than the fEPSP amplitude, it is an obvious limitation of the present study that we cannot distinguish between Kv7 on Schaffer collateral terminals and GABAergic interneurons. If we assume that XE991 increases GABA release, this compound should decrease PTP but increase PPR. This implies that at least the decreased PPR in epileptic CA1 could be one putative consequence of such an XE991 effect on GABAergic interneurons. Another limitation may be deduced from the aforementioned study on postsynaptic mechanisms involved in PPR changes after tetanic stimulation [38]. While the present study cannot exclude such a postsynaptic involvement, our findings from XE991-treated epileptic tissue with obviously less Kv7 channel expression [17,18,19] argue against a major impact of this condition.

In the absence of XE991, PTP was associated with a significant drop in the PPR in both control and epileptic slices. The most parsimonious explanation for this drop is that increased presynaptic calcium following tetanic stimulation had strengthened release probability and thereby reduced the PPR. Blocking presynaptic Kv7 channels should also increase presynaptic calcium and thus increase transmitter release [14,40] with a subsequent drop of PPR. In fact, XE991 itself had depressed the PPR—measured with fEPSP amplitudes—in both control and epileptic [19]. Here, using fEPSP slopes instead of amplitudes, XE991 led to a PPR drop in epileptic slices only, while the slight PPR reduction in control slices was not statistically significant. Hence, both studies taken together found the consistent finding of a reduced PPR in epileptic slices, which was much less profound in control slices. Therefore, we suggest that the impact of Kv7 channels on transmitter release at Schaffer collateral–CA1 synapses in epilepsy is probably higher than under control conditions.

How may potentiation in general be altered in epileptic slices? As discussed above, tetanic stimulation leads to a presynaptic calcium increase and, in turn, PTP [28,29]. If we assume that epileptic tissue exhibits a more prevalent presynaptic Kv7 channel function, this should reduce release probability under basal conditions, and tetanic stimulation should produce a stronger increase of presynaptic calcium. Both of these consequences would explain the higher PPR at baseline and the higher PTP levels in the absence of XE991. When, however, release probability is already enhanced—as in the presence of XE991—there might be less potential of tetanic stimulation to further increase it. This would explain why there was no significant PPR drop following tetanic stimulation in XE991-treated epileptic slices. The higher PTP level in epileptic slices in the absence of XE991 also implies a higher propensity to exhibit long-term potentiation (LTP) in these slices. In fact, we demonstrated higher theta-burst LTP levels after pilocarpine-induced status epilepticus, due to a persistent GluN2B upregulation in chronically epileptic CA1 [24]. Since this upregulation was presumably postsynaptic, we now conclude that the enhanced propensity of epileptic Schaffer collateral–CA1 synapses to potentiate follows the enhanced function of both postsynaptic GluN2B and presynaptic Kv7.

What mechanisms could also contribute to the XE991-related normalization of short-term plasticity in epileptic tissue? As discussed above, XE991 targets other than presynaptic Kv7 channels on Schaffer collateral terminals include interneurons and GABAergic signaling in control tissue [14,40,41,42,43]. However, with respect to epileptic tissue, a recent excellent study using human neuronal–astrocytic co-cultures showed that Kv7.2 loss-of-function mutations not only induced hyperexcitability but also led to long-term transcriptional changes [45]. The consequence was a maladaptively remodeled co-culture, and we may speculate whether such a condition could have been recapitulated in the context of the epilepsy-related Kv7.2 downregulation [17,18,19]. Although future studies will be needed to shed more light on long-term sequelae of epilepsy-associated Kv7 malfunction, it is conceivable that XE991 exerted additional network-level effects in epileptic tissue which are absent in control.

## 5. Conclusions

The present study revealed enhanced short-term plasticity in the epileptic CA1 area. Importantly, this was reversed by pre-application with XE991. Given the typical presynaptic origin of short-term plasticity, our findings add further evidence that presynaptic Kv7 channels are preserved in the epileptic CA1. Moreover, our data point to an increased rather than decreased presynaptic Kv7 function at Schaffer collateral–CA1 synapses. This finding could potentially be clinically relevant since it generates the following pathophysiological hypothesis: While Kv7-specific potassium channel activators may be beneficial in epileptic tissue with reduced postsynaptic Kv7 channel function, it may in turn entail a risk of further enhancing short-term plasticity in the same tissue.

## Figures and Tables

**Table 1:** Overview of all *p* values of 2-way ANOVA for slice-based and for animal-based conditions. Two-way ANOVA was calculated using the factor animal (“Ctrl” versus “Epil”), the factor drug (“XE” versus “no XE”), and the interaction animal × drug. When applicable, the Holm–Sidak post hoc test was used for all pairwise comparisons. All data are given as the mean (SEM).

Parameter	Group	Data	*p* Value (2-Way ANOVA)	*p* Value (Holm–Sidak Post Hoc)
**Slice-based analysis**				
fEPSP slope (timepoint ptp) in % of pre	Ctrl	173 (12)	Animal: 0.434	Drug within “Ctrl”: 0.769
	Ctrl-XE	167 (12)	Drug: 0.004	Drug within “Epil”: <0.001
	Epil	219 (19)	Animal × Drug: 0.013	Animal within “no XE”: 0.023
	Epil-XE	142 (6)		Animal within “XE”: 0.201
fEPSP slope (timepoint post) in % of pre	Ctrl	171 (15)	Animal: 0.154	Drug within “Ctrl”: 0.99
	Ctrl-XE	171 (11)	Drug: 0.007	Drug within “Epil”: <0.001
	Epil	242 (26)	Animal × Drug: 0.007	Animal within “no XE”: 0.005
	Epil-XE	148 (8)		Animal within “XE”: 0.331
fEPSP amplitude (timepoint pre) in mV	Ctrl	−1.02 (0.09)	Animal: <0.001	Not applicable
	Ctrl-XE	−1.00 (0.07)	Drug: 0.647	
	Epil	−0.70 (0.09)	Animal × Drug: 0.877	
	Epil-XE	−0.65 (0.07)		
PPR (timepoint pre)	Ctrl	1.31 (0.10)	Animal: 0.231	Drug within “Ctrl”: 0.579
	Ctrl-XE	1.14 (0.11)	Drug: 0.003	Drug within “Epil”: <0.001
	Epil	1.73 (0.15)	Animal × Drug: 0.022	Animal within “no XE”: 0.016
	Epil-XE	1.07 (0.11)		Animal within “XE”: 0.409
**Animal-based analysis**				
fEPSP slope (timepoint ptp) in % of pre	Ctrl	170 (13)	Animal: 0.418	Drug within “Ctrl”: 0.965
	Ctrl-XE	169 (10)	Drug: 0.021	Drug within “Epil”: 0.005
	Epil	223 (18)	Animal × Drug: 0.023	Animal within “no XE”: 0.032
	Epil-XE	141 (7)		Animal within “XE”: 0.222
fEPSP slope (timepoint post) in % of pre	Ctrl	170 (14)	Animal: 0.163	Drug within “Ctrl”: 0.974
	Ctrl-XE	169 (10)	Drug: 0.027	Drug within “Epil”: 0.007
	Epil	250 (31)	Animal × Drug: 0.029	Animal within “no XE”: 0.027
	Epil-XE	148 (6)		Animal within “XE”: 0.479
fEPSP amplitude (timepoint pre) in mV	Ctrl	−1.04 (0.08)	Animal: 0.005	Not applicable
	Ctrl-XE	−0.98 (0.09)	Drug: 0.49	
	Epil	−0.72 (0.07)	Animal × Drug: 0.994	
	Epil-XE	−0.65 (0.02)		
PPR (timepoint pre)	Ctrl	1.28 (0.13)	Animal: 0.167	Not applicable
	Ctrl-XE	1.13 (0.08)	Drug: 0.017	
	Epil	1.78 (0.19)	Animal × Drug: 0.105	
	Epil-XE	1.08 (0.10)		

## Data Availability

Research data are stored in an institutional repository and will be shared upon reasonable request to the corresponding author.

## Abbreviations

The following abbreviations are used in this manuscript:

fEPSP	Field excitatory postsynaptic potential
GluN2B	Glutamate receptor, NMDA, type 2B
LTP	Long-term potentiation
PPR	Paired-pulse ratio
PTP	Post-tetanic potentiation
STP	Short-term potentiation

## References

[1] Yue C., Yaari Y. (2004). KCNQ/M channels control spike afterdepolarization and burst generation in hippocampal neurons. J. Neurosci..

[2] Gu N., Vervaeke K., Hu H., Storm J.F. (2005). Kv7/KCNQ/M and HCN/h, but not K_Ca_2/SK channels, contribute to the somatic medium after-hyperpolarization and excitability control in CA1 hippocampal pyramidal cells. J. Physiol..

[3] Brown J.T., Randall A.D. (2009). Activity-dependent depression of the spike after-depolarization generates long-lasting intrinsic plasticity in hippocampal CA3 pyramidal neurons. J. Physiol..

[4] Petrovic M.M., Nowacki J., Olivo V., Tsaneva-Atanasova K., Randall A.D., Mellor J.R. (2012). Inhibition of post-synaptic Kv7/KCNQ/M channels facilitates long-term potentiation in the hippocampus. PLoS ONE.

[5] Song M.-K., Cui Y.-Y., Zhang W.-W., Zhu L., Lu Y., Chen H.-Z. (2009). The facilitating effect of systemic administration of Kv7/M channel blocker XE991 on LTP induction in the hippocampal CA1 area independent of muscarinic activation. Neurosci. Lett..

[6] Suzuki E., Okada T. (2012). Stratum oriens stimulation-evoked modulation of hippocampal long-term potentiation involves the activation of muscarinic acetylcholine receptors and the inhibition of Kv7/M potassium ion channels. Eur. J. Neurosci..

[7] Laker D., Tolle F., Stegen M., Heerdegen M., Köhling R., Kirschstein T., Wolfart J. (2021). Kv7 and Kir6 Channels Shape the Slow AHP in Mouse Dentate Gyrus Granule Cells and Control Burst-like Firing Behavior. Neuroscience.

[8] Soderling T.R., Derkach V.A. (2000). Postsynaptic protein phosphorylation and LTP. Trends Neurosci..

[9] Yue C., Yaari Y. (2006). Axo-somatic and apical dendritic Kv7/M channels differentially regulate the intrinsic excitability of adult rat CA1 pyramidal cells. J. Neurophysiol..

[10] Cooper E.C., Harrington E., Jan Y.N., Jan L.Y. (2001). M channel KCNQ2 subunits are localized to key sites for control of neuronal network oscillations and synchronization in mouse brain. J. Neurosci..

[11] Geiger J., Weber Y.G., Landwehrmeyer B., Sommer C., Lerche C. (2006). Immunohistochemical analysis of KCNQ3 potassium channels in mouse brain. Neurosci. Lett..

[12] Weber Y.G., Geiger J., Kampchen K., Landwehrmeyer B., Sommer C., Lerche C. (2006). Immunohistochemical analysis of KCNQ2 potassium channels in adult and developing mouse brain. Brain Res..

[13] Shah M.M., Migliore M., Valencia I., Cooper E.C., Brown D.A. (2008). Functional significance of axonal Kv7 channels in hippocampal pyramidal neurons. Proc. Natl. Acad. Sci. USA.

[14] Peretz A., Sheinin A., Yue C., Degani-Katzav N., Gibor G., Nachman R., Gopin A., Tam E., Shabat D., Yaari Y. (2007). Pre- and postsynaptic activation of M-channels by a novel opener dampens neuronal firing and transmitter release. J. Neurophysiol..

[15] Biervert C., Schroeder B.C., Kubisch C., Berkovic S.F., Propping P., Jentsch T.J., Steinlein O.K. (1998). A potassium channel mutation in neonatal human epilepsy. Science.

[16] Singh N.A., Charlier C., Stauffer D., DuPont B.R., Leach R.J., Melis R., Ronen G.M., Bjerre I., Quattlebaum T., Murphy J.V. (1998). A novel potassium channel gene, KCNQ2, is mutated in an inherited epilepsy of newborns. Nat. Genet..

[17] Maslarova A., Salar S., Lapilover E., Friedman A., Veh R.W., Heinemann U. (2013). Increased susceptibility to acetylcholine in the entorhinal cortex of pilocarpine-treated rats involves alterations in KCNQ channels. Neurobiol. Dis..

[18] Zhu C., Lin R., Liu C., Huang M., Lin F., Zhang G., Zhang Y., Miao J., Lin W., Huang H. (2020). The Antagonism of 5-HT6 Receptor Attenuates Current-Induced Spikes and Improves Long-Term Potentiation via the Regulation of M-Currents in a Pilocarpine-Induced Epilepsy Model. Front. Pharmacol..

[19] Müller S., Kartheus M., Hendinger E., Hübner D.C., Schnell E., Rackow S., Bertsche A., Köhling R., Kirschstein T. (2024). Persistent Kv7.2/7.3 downregulation in the rat pilocarpine model of mesial temporal lobe epilepsy. Epilepsy Res..

[20] Su Z., Li Y., Chen S., Liu X., Zhao K., Peng Y., Zhou L. (2022). Identification of Ion Channel-Related Genes and miRNA-mRNA Networks in Mesial Temporal Lobe Epilepsy. Front. Genet..

[21] Nissen E.M., Kirschstein T., Bertsche A., Köhling R., Franz D. (2026). Loose-patch recordings reveal differential Kv7-dependent effects on spontaneous excitatory activity in control and epileptic CA1. Front. Neurol..

[22] Turski W.A., Cavalheiro E.A., Schwarz M., Czuczwar S.J., Kleinrok Z., Turski L. (1983). Limbic seizures produced by pilocarpine in rats: Behavioural, electroencephalographic and neuropathological study. Behav. Brain Res..

[23] Racine R.J. (1972). Modification of seizure activity by electrical stimulation. II. Motor seizure. Electroencephalogr. Clin. Neurophysiol..

[24] Müller L., Tokay T., Porath K., Köhling R., Kirschstein T. (2013). Enhanced NMDA receptor-dependent LTP in the epileptic CA1 area via upregulation of NR2B. Neurobiol. Dis..

[25] Postnikova T.Y., Zubareva O.E., Kovalenko A.A., Kim K.K., Magazanik L.G., Zaitsev A.V. (2017). Status Epilepticus Impairs Synaptic Plasticity in Rat Hippocampus and Is Followed by Changes in Expression of NMDA Receptors. Biochemistry.

[26] Gholami M., Hosseinmardi N., Mirnajafi-Zadeh J., Javan M., Semnanian S., Naghdi N., Fathollahi Y. (2020). Long-term potentiation enhancing effect of epileptic insult in the CA1 area is dependent on prior-application of primed-burst stimulation. Exp. Brain Res..

[27] Salaka R.J., Nair K.P., Sasibhushana R.B., Udayakumar D., Kutty B.M., Srikumar B.N., Rao B.S.S. (2022). Differential effects of levetiracetam on hippocampal CA1 synaptic plasticity and molecular changes in the dentate gyrus in epileptic rats. Neurochem. Int..

[28] Kuhnt U., Voronin L.L. (1994). Interaction between paired-pulse facilitation and long-term potentiation in area CA1 of guinea-pig hippocampal slices: Application of quantal analysis. Neuroscience.

[29] Schulz P.E., Cook E.P., Johnston D. (1994). Changes in paired-pulse facilitation suggest presynaptic involvement in long-term potentiation. J. Neurosci..

[30] Rosahl T.W., Geppert M., Spillane D., Herz J., Hammer R.E., Malenka R.C., Südhof T.C. (1993). Short-term synaptic plasticity is altered in mice lacking synapsin I. Cell.

[31] Geppert M., Goda Y., Stevens C.F., Südhof T.C. (1997). The small GTP-binding protein Rab3A regulates a late step in synaptic vesicle fusion. Nature.

[32] Schoch S., Castillo P.E., Jo T., Mukherjee K., Geppert M., Wang Y., Schmitz F., Malenka R.C., Südhof T.C. (2002). RIM1alpha forms a protein scaffold for regulating neurotransmitter release at the active zone. Nature.

[33] Ferguson G.D., Wang H., Herschman H.R., Storm D.R. (2004). Altered hippocampal short-term plasticity and associative memory in synaptotagmin IV (−/−) mice. Hippocampus.

[34] Breustedt J., Gundlfinger A., Varoqueaux F., Reim K., Brose N., Schmitz D. (2010). Munc13-2 differentially affects hippocampal synaptic transmission and plasticity. Cereb. Cortex.

[35] López-Murcia F.J., Krueger-Burg D., Wenger S., López-Hernández T., Lipstein N., Taschenberger H., Brose N. (2026). Ca^2+^-phospholipid-dependent regulation of Munc13-1 is essential for post-tetanic potentiation at mossy fiber synapses and supports working memory. Cell Rep..

[36] Giansante G., Marte A., Romei A., Prestigio C., Onofri F., Benfenati F., Baldelli P., Valente P. (2020). Presynaptic L-Type Ca^2+^ Channels Increase Glutamate Release Probability and Excitatory Strength in the Hippocampus during Chronic Neuroinflammation. J. Neurosci..

[37] Louis-Gray K.R., Beatty J.A., Cox C.L. (2025). A novel mechanism for short-term post-tetanic plasticity in thalamocortical neurons. Brain Res..

[38] Wang J.H., Kelly P.T. (1997). Attenuation of paired-pulse facilitation associated with synaptic potentiation mediated by postsynaptic mechanisms. J. Neurophysiol..

[39] Sheridan G.K., Moeendarbary E., Pickering M., O’Connor J.J., Murphy K.J. (2014). Theta-burst stimulation of hippocampal slices induces network-level calcium oscillations and activates analogous gene transcription to spatial learning. PLoS ONE.

[40] Martire M., Castaldo P., D’Amico M., Preziosi P., Annunziato L., Taglialatela M. (2004). M channels containing KCNQ2 subunits modulate norepinephrine, aspartate, and GABA release from hippocampal nerve terminals. J. Neurosci..

[41] Grigorov A., Moskalyuk A., Kravchenko M., Veselovsky N., Verkhratsky A., Fedulova S. (2014). Kv7 potassium channel subunits and M currents in cultured hippocampal interneurons. Pflügers Arch..

[42] Pérez-Ramírez M.B., Laville A., Tapia D., Duhne M., Lara-González E., Bargas J., Galarraga E. (2015). Kv7 Channels Regulate Firing during Synaptic Integration in GABAergic Striatal Neurons. Neural Plast..

[43] Margineanu D.G., Wülfert E. (2000). Differential paired-pulse effects of gabazine and bicuculline in rat hippocampal CA3 area. Brain Res. Bull..

[44] Kuenzi F.M., Fitzjohn S.M., Morton R.A., Collingridge G.L., Seabrook G.R. (2000). Reduced long-term potentiation in hippocampal slices prepared using sucrose-based artificial cerebrospinal fluid. J. Neurosci. Methods.

[45] Dirkx N., Kaji M., De Vriendt E., Carleo G., Miceli F., Asselbergh B., Verstraelen P., Zonnekein N., Carotenuto L., Dang L.T. (2026). Kv7.2 loss-of-function causes early hyperexcitability and network remodelling. Brain.

